# Characterisation of liver fat in the UK Biobank cohort

**DOI:** 10.1371/journal.pone.0172921

**Published:** 2017-02-27

**Authors:** Henry R. Wilman, Matt Kelly, Steve Garratt, Paul M. Matthews, Matteo Milanesi, Amy Herlihy, Micheal Gyngell, Stefan Neubauer, Jimmy D. Bell, Rajarshi Banerjee, E. Louise Thomas

**Affiliations:** 1 Perspectum Diagnostics, Oxford, United Kingdom; 2 Department of Life Sciences, University of Westminster, London, United Kingdom; 3 UK Biobank, Stockport, United Kingdom; 4 Division of Brain Sciences and Centre for Neurotechnology, Imperial College London, United Kingdom; 5 OCMR, Division of Cardiovascular Medicine, Radcliffe Department of Medicine, University of Oxford, Oxford, United Kingdom; Chang Gung Memorial Hospital Kaohsiung Branch, TAIWAN

## Abstract

Non-alcoholic fatty liver disease and the risk of progression to steatohepatitis, cirrhosis and hepatocellular carcinoma have been identified as major public health concerns. We have demonstrated the feasibility and potential value of measuring liver fat content by magnetic resonance imaging (MRI) in a large population in this study of 4,949 participants (aged 45–73 years) in the UK Biobank imaging enhancement. Despite requirements for only a single (≤3min) scan of each subject, liver fat was able to be measured as the MRI proton density fat fraction (PDFF) with an overall success rate of 96.4%. The overall hepatic fat distribution was centred between 1–2%, and was highly skewed towards higher fat content. The mean PDFF was 3.91%, and median 2.11%. Analysis of PDFF in conjunction with other data fields available from the UK Biobank Resource showed associations of increased liver fat with greater age, BMI, weight gain, high blood pressure and Type 2 diabetes. Subjects with BMI less than 25 kg/m^2^ had a low risk (5%) of high liver fat (PDFF > 5.5%), whereas in the higher BMI population (>30 kg/m^2^) the prevalence of high liver fat was approximately 1 in 3. These data suggest that population screening to identify people with high PDFF is possible and could be cost effective. MRI based PDFF is an effective method for this. Finally, although cross sectional, this study suggests the utility of the PDFF measurement within UK Biobank, particularly for applications to elucidating risk factors through associations with prospectively acquired data on clinical outcomes of liver diseases, including non-alcoholic fatty liver disease.

## Introduction

The UK Biobank imaging enhancements provide an ideal resource with which to investigate the UK prevalence of liver steatosis, non-alcoholic fatty liver disease (NAFLD) and associated pathologies. NAFLD is defined as an accumulation of fat in the liver (hepatic steatosis) in greater than 5% of liver tissue in the absence of other causes (e.g. alcohol consumption, steatogenic medication) [1,2]. Estimates of the prevalence of NAFLD in the UK and other populations vary; a large prospective study in the UK using ultrasound reported the incidence of NAFLD to be 26.4% [3]. A recent meta-analysis examining world-wide prevalence of NAFLD involving 8,515,431 subjects found that rates varied from 13.5–31.8% in different regions [4–6], while prevalence in the USA has been estimated to be between 30% [7,8] and 46% [9]. Other studies have shown that obese and morbidly obese populations are particularly affected, with between 70–91% of such patients suffering from NAFLD [10]. Although NAFLD alone is a concerning condition, its roles as a risk or causal factor for non-alcoholic steatohepatitis (NASH), hepatic cirrhosis or hepatocellular carcinoma (HCC) are arguably a greater worry [11]. The biological mechanisms for this progression from NAFLD still are not well understood.

Estimates as to the proportion of patients who progress from NAFLD to non-alcoholic steatohepatitis (NASH) vary widely. One reason for this is the absence of universally accepted definitions for both NAFLD and NASH [12]. Existing clinical definitions rely on histology which is invasive and expensive [13]. Histology can also be affected by sampling error and therefore is often falsely negative [14]. Although several non-invasive diagnostic tools have been proposed, they have limited agreement in diagnosing NAFLD and NASH [15], and have not replaced histology as the clinical standard. A second reason is that most studies investigate disease progression in clinical cohorts rather than in the general population. This approach may well overestimate the association and rate of progression, as these cohorts typically have more risk factors for NASH than in addition to NAFLD [6,16].

The prevalence and risk of progression of NASH is better understood. The worldwide prevalence of NASH is reported to be between 1.5–6.45% [6]. It is estimated that 21–26% of NASH sufferers progress to cirrhosis in 8.2 years [17] with 7% progressing to HCC in 6.5 years [12]. The presence of NASH carries a risk of advanced fibrosis of 68 per 1,000 person years [6].

NAFLD is linked to metabolic risk factors, and in particular type 2 diabetes. Hepatic insulin resistance and NASH precede systemic insulin resistance in the development of diabetes, so NAFLD is increasingly thought of as a precursor to type 2 diabetes [18]. One study reported 23% of patients with NAFLD, and 47% of those with NASH, also have diabetes (a similar prevalence of diabetes to that in obese subjects) [6]. Similarly, in patients with type 2 diabetes the prevalence of NAFLD has been reported as 50% [19]. The combination of diabetes and NAFLD is a risk factor for progression into NASH, cirrhosis and death [6]. NAFLD is clearly an important cofactor in metabolic disorders. However, the understanding of how NAFLD relates to the other features of metabolic disorders, and how it progresses to NASH and HCC has been limited. This is due to people only being screened when presenting with symptoms, and screened using the invasive and limited method of tissue biopsy. Magnetic resonance imaging (MRI) now provides a promising non-invasive diagnostic alternative that can be used for large scale population studies and for serial follow up of patients at risk.

MRI and magnetic resonance spectroscopy (MRS) are now considered “gold-standard” methods for quantitative fat measurement. Fat quantitative MRI methods, generally based on Dixon or IDEAL pulse sequences, where the combination of different images can give fat and water measurements [20–22]. MRS is considered the more robust and quantitative of the two MR-based methods [23,24]. It shows good agreement with MRI but tends to be more sensitive, particularly are very low levels of fat infiltration [25,26]; however, it is limited by technical demands and availability, and therefore has been applied to relatively small studies in specialist centres.

The UK Biobank is a rich resource for evaluating risk factors for later life chronic disease. It includes lifestyle, clinical, biomarker and genomic data collected from a cohort of 500,000 UK individuals, aged between 40–69 years. In depth phenotyping based on a variety of imaging techniques, including MRI, DEXA and ultrasound now is being carried out in a subset of the participants (intended to include 100,000 by about 2022) [27]. The range of information available within the cohort provides an excellent opportunity to explore complex interaction of liver fat with both liver-related outcomes, and metabolic disease. In particular, as the imaged cohort increases in size, and the longer term patient outcome data becomes available, it will provide a dataset with which to develop risk profiles for a range of liver related outcomes based on liver biomarkers, as well as other biomarkers derived from genetic, epigenetic and lifestyle factors. In this pilot study, we have investigated the prevalence of NAFLD in the UK Biobank cohort for the first time and assessed the potential impact of age, gender and BMI on hepatic fat deposition.

## Methods

### Study design

A prospective study of 4949 subjects from the UK Biobank imaging enhancement protocol [27,28] acquired between August 2014 and October 2015. These 4949 subjects were those that were scanned chronologically first and have had their scans made available from the UK Biobank imaging enhancements, with no inclusion or exclusion criteria in addition to those used by the UK Biobank imaging enhancements. Proton density fat fraction (PDFF) and patient meta-data were obtained through UK Biobank Access Application number 9914. The UK Biobank has approval from the North West Multi-Centre Research Ethics Committee (MREC), and obtained written informed consent from all participants prior to the study.

### Imaging protocol

Images were acquired at the dedicated UK Biobank imaging Centre at Cheadle (UK) using a Siemens 1.5T MAGNETOM Aera. A multi-echo spoiled-gradient-echo acquisition was used to calculate T2* and PDFF maps of the liver [20,21]. A single transverse slice was captured, through the centre of the liver superior to the porta hepatis. The following parameters were used: 40x40cm^2^ field of view, 160x160 acquisition matrix yielding a voxel size of 2.5mm x 2.5mm, 6mm slice thickness, 20° flip angle, 27ms TR, and 2 signal averages. Ten echo times were selected such that the signals from fat and water were in phase and out of phase at 1.5T (TE = 2.38, 4.76, 7.14, 9.52, 11.90, 14.28, 16.66, 19.04, 21.42, and 23.80 ms). The acquisition of echoes needed for PDFF image construction occurred during a single expiration breath-hold, without any contrast agent, and typically took ≤ 3 minutes.

### Post-processing

Raw MR images were sent to a central reporting laboratory, before being transferred to individual Windows 7 workstations for analysis.

### Image analysis

MR data were analysed using Liver*MultiScan*^*™*^ Discover software [29]. PDFF maps were constructed using the second, fourth, and sixth of the ten MR echoes, using a three-point DIXON technique [20,21]. Image analysts were trained in abdominal anatomy, to allow them to reject images where the liver was not clearly identifiable. Image analysts were also trained in common MR artefacts, allowing them to identify artefacts within the images, such as poor shimming, motion, low signal, and fat swaps. Images with artefacts were referred to a team of experienced MR physicists for evaluation. Fat-water swaps were corrected by subtracting the measured PDFF value from 100%.

Three 15mm diameter circular regions of interest (ROIs) were selected on the PDFF images. ROIs were selected to cover a representative sample of the liver parenchyma, avoiding vessels, bile ducts and other organs. The reported PDFF was calculated from the mean of all of the pixels within the three ROIs. Scans were anonymised and thus analysts were blinded to the meta-data about each participant.

### Inter- and intra-reader Variability

Inter- and Intra-reader variability has been previously reported [30]. Over different days, the mean intra-reader correlation was 0.96 (SD = 0.01), and the mean inter-reader correlation was 0.997 (SD = 0.0001).

### Statistical analysis

Summary data are presented as medians and quartiles. Comparison between groups was tested using Kruskal-Wallis and two-sided Kolmogorov-Smirnov (KS) tests. Due to ties in the data, p-values are approximated for the KS tests, with 2e-16 the lowest value that can be accurately reported. A linear model was constructed using a natural log transformation to normalise the fat distribution. The performance of the of the model was evaluated by analysis of residual plots, and calculating the correlation between the parameter estimates and the variance inflation factors [31]. Statistical analyses were undertaken using Python 2.7 [32] and R 3.1.1 [33].

## Results

**Fig 1:**
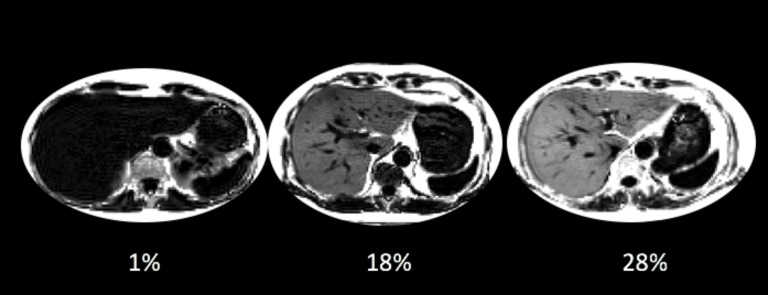
Representative MRI images from the UKBiobank cohort, showing individuals with 1%, 18% and 28% PDFF. Clear differences in the intensity of the liver can be seen, with the liver appearing brighter as PDFF increases.

PDFF was successfully calculated from 4775 (96.8%) of the initial 4949 MRI datasets. Of the 174 images not used, 88 were rejected during a quality control process because of missing files, 44 had artefacts, in 21 cases the slice was incorrectly positioned such that it did not include the liver and 19 files were corrupted not load. A further 158 (3.31%) could not be linked to meta-data. This left a total of 4617 datasets (for example MRI images, see Fig 1). 18 of these datasets had fat-swaps. 251 datasets had missing meta-data in one or more of the columns described in Table 1, but these have been included in individual analysis where relevant data were available.

**Table 1:** Details of UKBiobank data fields used in this analysis.

Field	Field ID	Instance	Short name
Sex	31	NA	Sex
Body mass index (BMI)	21001	2 –Imaging visit	BMI
Diabetes diagnosed by doctor	2443	2 –Imaging visit	Diabetes
Vascular/heart problems diagnosed by doctor	6150	2 –Imaging visit	Vascular/heart problems
Alcohol drinker status	20117	2 –Imaging visit	Alcohol drinker status
Ethnic background	21000	2 –Imaging visit	Ethnic background
Weight change compared with 1 year ago	100540	2 –Imaging visit	One year weight change
Age when attended assessment centre	21003	2 –Imaging visit	Date of attending assessment centre

The demographics of the subjects included in the final dataset are described in Tables 2 and 3. Overall, the cohort was approximately balanced for gender (female: male ratio 53: 47), with a median BMI of 26.09 kg/m^2^ (range 16.04–48.84 kg/m^2^), making this cohort marginally ‘overweight’ (>25 kg/m^2^) on average. The age range was 45–73 years, centred around a median of 62 years; more than 80% of the cohort was aged between 50–70 years. The cohort was predominantly European ethnicity, with 95.8% reporting a white ethnicity.

**Table 2:** Cohort demographics.

	Subjects (n)	Subjects (%)
**Gender**		
Male	2184	47.3
Female	2433	52.7
**Age**		
40–49 yrs	334	7.2
50–59 yrs	1371	29.7
60–69 yrs	2146	46.5
70–79 yrs	567	12.3
Unavailable	199	4.3
**BMI (kg/m**^**2**^**)**		
<20 kg/m^2^	133	2.9
20–25 kg/m^2^	1646	35.7
25–30 kg/m^2^	1952	42.3
30–35 kg/m^2^	657	14.2
>35 kg/m^2^	209	4.5
Unavailable	20	0.4
**Ethnicity**		
White	4421	95.8
Mixed	28	0.6
Asian	52	1.1
Black	29	0.6
Chinese	13	0.3
Other	21	0.5
Unavailable	53	1.1
**Vascular/heart problems**		
Angina	108	2.3
Heart attack	98	2.1
High blood pressure	1239	26.8
Stroke	58	1.3
None of the above	3204	69.4
Unavailable	52	1.1
**Diabetes**		
Yes	226	4.9
No	4336	93.9
Unavailable	55	1.2
**Alcoholic drinker status**		
Current	4263	92.3
Previous	160	3.5
Never	148	3.2
Unavailable	46	1.0
**Self-reported weight change**		
Yes—loss	865	18.7
No	2635	57.1
Yes—gain	1017	22.0
Unavailable	100	2.2

The ‘unavailable’ rows include missing values, ‘don’t know’, and ‘prefer not to say’ responses. For vascular/heart problems, subjects could have any number of labels (i.e. they could be in both the heart attack and stroke groups). For all other phenotype, subjects could only have a single label.

**Table 3:** Age and body mass index summary (BMI) statistics of cohort.

	Median	IQR	1^st^ Quartile	3^rd^ Quartile	Min value	Max value	Mean
Age (years)	62	12	55	67	45	73	61.19
BMI (kg/m^2^)	26.09	5.34	23.61	28.95	16.04	48.84	26.65

**Fig 2:**
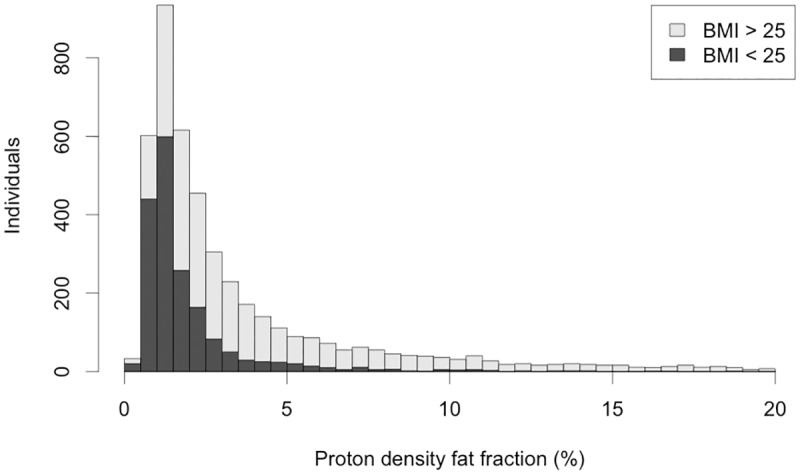
Distribution of PDFF in whole cohort. There are 84 individuals with PDFF > 20%, who are not shown here. The darker part of each bar corresponds to the individuals with BMI > 25 kg/m^2^.

The maximum of the overall hepatic fat distribution (Fig 2) was centred between 1–2%, and was highly skewed towards higher fat content. The mean PDFF was 3.91%, and median 2.11% (IQR 1.25–4.37). 19.9% of individuals (n = 921) had >5.5% PDFF, the commonly accepted risk level for NAFLD [1], and 9.2% of the participants (n = 425) had a PDFF > 10%.

### BMI

**Fig 3:**
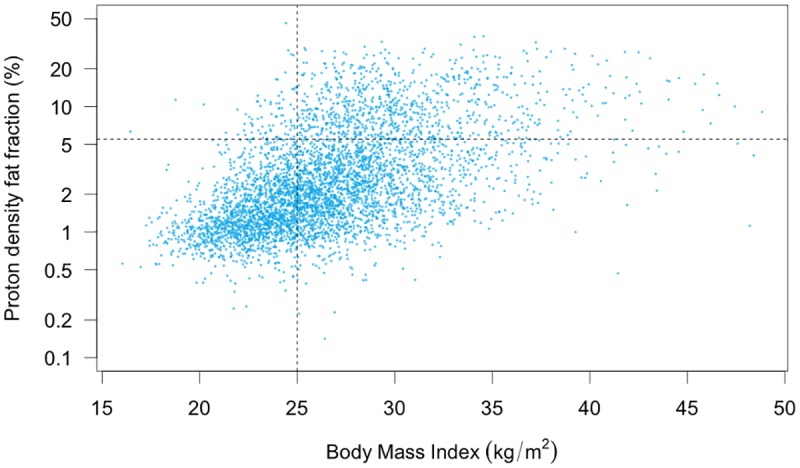
Relationship between proton density fat fraction and body mass index. PDFF is plotted on a log scale. 83 (1.8%) individuals are in the top left quadrant, 835 (18.1%) in the top right, 1983 (42.9%) in the bottom right and 1696 (36.7%) in the bottom left quadrant.

The upper end of the distribution was dominated by those with high BMI, with 90% of those participants who had PDFF >5% having BMI > 25 kg/m^2^. By comparison, ‘healthy’ subjects in the imaged cohort (BMI < 25 kg/m^2^, no diabetes diagnosed by doctor), had lower PDFF values, (mean 1.97%, median 1.32%, IQR 0.99–2.06%). In addition, this subset had a smaller spread, with standard deviation 2.33 and IQR 1.07 (Table 4). The 95% upper limit for ‘healthy’ participants was 5.21%. Our results showed that there is clearly a strong correlation between higher liver fat and BMI (Fig 3). PDFF distributions of the cohort split into brackets of 5 BMI units, showed a steady increase in median PDFF (less than 20 kg/m^2^: 0.98%, 20–25 kg/m^2^: 1.36%, 25–30 kg/m^2^: 2.54%, 30–35 kg/m^2^: 4.39%, and more than 35 kg/m^2^: 7.33%, see S1 Table). These data suggest that BMI can be used to stratify subjects in this population for risk of a high PDFF; only 83 subjects (1.8%) had both a BMI less than 25 kg/m^2^ and a PDFF greater than 5.5% (Fig 3).

**Table 4:** Summary statistics for whole cohort PDFF and for subset with 'healthy' BMI.

	Mean	St. dev	5^th^ Percentile	1^st^ Quartile	Median	3^rd^ Quartile	95^th^ Percentile
Whole cohort	3.91%	4.64%	0.78%	1.25%	2.11%	4.37%	14.01%
BMI < 25 kg/m^2^ and no Diabetes	1.97%	2.33%	0.68%	1.00%	1.32%	2.05%	5.21%

### Gender and age

**Fig 4:**
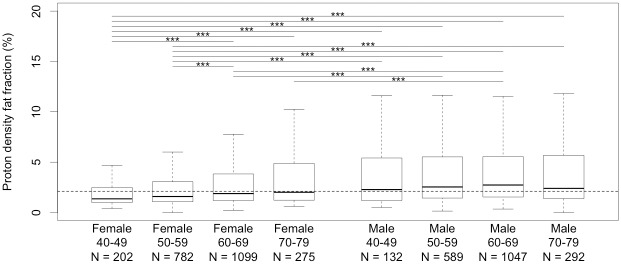
Distribution of liver fat by age group and sex. The dashed line shows the population median PDFF (2.11%). *** indicates p < 0.001 from a two-sided K-S test. Significance lines are only shown for p <0.001.

We also observed a strong relationship between liver fat, gender and age (Table 5 and Fig 4). Females (median 1.76%, IQR 1.14–3.54) had significantly lower liver fat than males (median 2.58%, IQR 1.48–5.47) (one-sided K-S test, p = 10^−34^). For the whole cohort, there was an increase in liver fat with age, although this was more strongly present in the female participants than the males. In females, the 40–49 year olds (median 1.36% IQR 0.98–2.46) had significantly lower PDFF than the 60–69 year olds (median 1.88%, IQR 1.20–3.82, p = 2.4e-6) and the 70–79 year olds (median 2.01%, IQR 1.21–4.48, p = 1.0e-5). Similarly, the 50–59 year olds (median 1.57%, IQR 1.06–2.92) had significantly lower PDFF than the 60–69 year olds (median 1.88%, IQR 1.20–3.82, p = 1.3e-4) and the 70–79 year olds (median 2.01%, IQR 1.21–4.48, p = 2.1e-3). The 40–49 and 50–59 year-old female groups had significantly lower PDFF values than all of the male age groups (all p-values in range 4e-4 – 1e-27). Conversely, there was no significant difference between the distribution of PDFF in any of the male age groups (40–49 years, 50–59 years, 60–69 years and 70–79 years), with all p-values in the range 0.24–0.054 (two-sided K-S tests). Despite differences in the female and male distributions as a whole, the differences became smaller and less significant as individuals age. In the 70–79 year-olds, although there was a difference in the medians between females (2.01%, IQR 1.21–4.48) and males (2.44%, IQR 1.36–5.45), their distributions are not significantly different (p-value 0.16). In comparison, the 50–59 year-old groups have median PDFFs of 1.57% (IQR 1.06–2.92) and 2.54% (IQR 1.42–5.41), and had significantly different distributions (p-value 1.3e-18).

**Table 5:** Cohort summary divided by age and sex.

Female	40–49 yrs	50–59 yrs	60–69 yrs	70–79 yrs	All
Median PDFF	1.36 (0.98–2.46)	1.57 (1.06–2.92)	1.88 (1.20–3.82)	2.01 (1.21–4.48)	1.76 (1.14–3.54)
Count	202	782	1099	275	2433
Male	40–49 yrs	50–59 yrs	60–69 yrs	70–79 yrs	All
Median PDFF	2.28 (1.20–5.40)	2.49 (1.42–5.41)	2.68 (1.54–5.52)	2.44 (1.36–5.45)	2.58 (1.48–5.47)
Count	132	589	1047	292	2184
All	40–49 yrs	50–59 yrs	60–69 yrs	70–79 yrs	All
Median PDFF	1.61 (1.07–3.17)	1.93 (1.17–3.97)	2.22 (1.33–4.57)	2.19 (1.27–4.72)	2.33 (1.21–4.10)
Count	334	1371	2146	567	4617

Median PDFFs are given with 1^st^ and 3^rd^ quartiles in brackets.

### Other metabolic phenotypes

**Fig 5:**
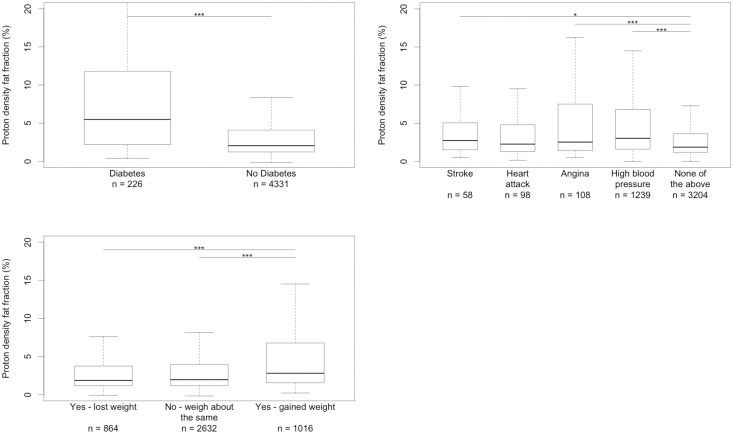
Box plots showing the distribution of PDFF for groups divided by several self-reported factors. Top left—Diabetes. Top right—Vascular problems. Bottom left—self-reported weight change over the year prior to imaging visit. *** indicates p < 0.001 and ** indicates p < 0.01 from a two-sided K-S test.

Diabetes, alcohol consumption, cardiovascular disease, and weight change are all factors that affect, or are affected by, metabolic syndromes. Liver fat was associated with self-reported diabetes, weight loss and vascular disease (Fig 5). There was no clear relation between self reported overall alcohol consumption and liver fat (p = 0.68, Kruskal-Wallis rank sum test).

All the groups that reported one of the cardiovascular problems had slightly higher PDFF than those who did not. However, there were only three groups with a significant difference from the group that reported no cardiovascular problems. Subjects with high blood pressure (median 3.03%, IQR 1.62–6.83) had the most different distribution (p < 10^−16^, two sided KS test) to those who reported no cardiovascular illness (median 1.87%, IQR 1.18–3.62), while the angina (median 2.53%, IQR 1.42–7.45) group had a less significant difference (p = 0.00012). The stroke group (median 2.73%, IQR 1.51–4.98) although statistically significant (p = 0.013) is unlikely to meet the threshold for significance after correction for multiple comparisons.

In terms of self reported weight change, the group reporting an increase in weight over the last year (median 2.81%, IQR 1.56–6.72) had significantly higher liver fat than the group reporting no change (p = 1.e-16) or loss of weight over the last year (p = 5.3e-13). However, there was no significant difference (p = 0.39) between the PDFF distributions in the group who reported no change (median 1.97%, IQR 1.19–3.97) and the group who reported a loss of weight (median 1.88%, IQR 1.18–3.78).

In addition, there was a significant difference between the groups that reported having been diagnosed with diabetes (median 5.49%, IQR 2.20–11.72), and those who do not (median 2.05%, IQR 1.23–4.10, p < 10^−16^, two sided KS test).

Combining all of these individually significant features (gender, age, BMI, high blood pressure, angina, weight gain, and diabetes) into a single linear model, with a log transformation of PDFF, yielded all but angina as significant features in the model (all p-values < 1.6 x10^-6^). S2 Table shows the regression coefficients, with BMI showing the most significant effect, followed by gender, diabetes, high blood pressure, age and finally self-reported weight gain. As PDFF was log transformed, regression coefficients relate to a relative increase in PDFF, so here a 1.10 times increase means a change in PDFF from 5–5.5% or 10–11%. The model suggested that men have 1.21 times (95% CI: 1.16–1.26) greater PDFF than women. An increase in a decade of age resulted in 1.071 times (95% CI: 1.040–1.103) the PDFF. Diabetes resulted in 1.42 times (95% CI: 1.29–1.57) the PDFF, and an increase in one BMI unit increased PDFF by a factor of 1.099 (95% CI: 1.093–1.104). According to this model, a liver fat of 2% at BMI 20 kg/m^2^ would increase to 5.13% liver fat, at BMI 30 kg/m^2^. These examples should be treated with caution, however, as the model assumes that all the correlations are linear with log transformed PDFF. The r-squared value for the linear model is 0.32, indicating that these factors explain less than half of the variation in PDFF.

Although all of these features (age, gender, BMI, self-reported weight loss, cardiovascular problems, diabetes) are correlated with liver fat, it is well known that they are also correlated with one another. However, analysis of the correlation of the variables (see S3 Table) revealed that the greatest correlation is 0.28 (between self-reported weight gain and BMI), and only two other pairs of variables have correlation greater than 0.15. Similar results were seen from analysis of the correlation of the regression coefficients. Furthermore, although the variance inflation factors [31] for each of the variables was greater than 1, BMI had the highest value, which is only 1.17, well below the suggested limit of 2.

Diabetes, and the combination of diabetes and high BMI, were clear risk factors for fatty liver—with positive predictive value (PPV) 0.500 (S4 Table) and 0.656 (S5 Table) respectively. However, the prevalence of both of these is small within the cohort. Although higher PPV could be obtained by using more of the predictors identified by the regression analysis, the number of individuals with a combination of these predictors was too small for useful conclusions to be drawn. BMI alone could be used to rule out high PDFF—with a negative predictive value (NPV) of 0.95 (Fig 3, S6 Table), however this did not have high specificity (0.46), and so has low PPV (0.30) for predicting high PDFF. Conversely, high BMI (> = 25kg/m^2^) and high PDFF (> = 5.5%) had a high sensitivity (0.87) for predicting the presence of Diabetes (S7 Table). Only 29 of the 226 individuals with diabetes did not have both elevated PDFF and elevated BMI.

## Discussion

This work demonstrates that the measurement of hepatic steatosis, by MRI-derived PDFF, on a large cohort is feasible and produces population level statistics relevant to epidemiology. The imaging process achieved 96.8% success rate. The MR sequence is fast (≤ 3 minutes), and requires no contrast agents.

The liver steatosis measurements from the Dallas heart study [1] has long been used to define the normal range of liver steatosis. The Dallas cohort was mixed in its ethnicity (50% Black, 30% White, 20% Hispanic), and younger than the UK Biobank (mean 46 years compared to mean 61 years in this study). The 95% upper limit of a healthy subset (BMI < 25 kg/m^2^, no diabetes or glucose intolerance, no excessive alcohol use and normal liver functions) of the Dallas study was 5.5%, compared to 5.21% in this UK Biobank cohort (BMI < 25 kg/m^2^, no diabetes). This indicates that using a steatosis level of 5.5% to identify at-risk subjects is a reasonable measure for the UK population aged 40–70 years. The UK Biobank will, in time, provide a good source of outcomes to further validate this number. The clear correlations observed here with age and sex indicate that it may be more appropriate to additionally define the ‘at risk’ threshold based on age and sex, rather than a single catch-all value of 5.5%

There was a clear correlation of liver steatosis with BMI, and BMI could be used clinically to stratify people for risk of fatty liver (NPV 0.95). Further analysis to identify characteristics of subsets of this ‘healthy’ population with clinically-significant hepatic steatosis is planned (e.g. individuals with low BMI and high steatosis), particularly if they correspond to the thin-outside, fat inside (‘TOFI’) phenotype [34]. The TOFI phenotype is characterised by low BMI, but high (or higher than expected) visceral fat.

There was a strong correlation between age and liver steatosis, as well as sex and steatosis. The age correlation was driven predominantly by the females within the cohort—the different male age groups do not show a significant difference. Menopause may have been the cause of the greater change in hepatic fat in females, in particular changes in estrogen levels or body fat percentage. The interaction of PDFF with other metabolic phenotypes is a reasonably complex pattern to tease apart—a linear model showed that all of age, sex and BMI have a significant effect on liver steatosis. It demonstrated that any further analysis of liver steatosis should account for these effects in their models. The effect of age is also complicated by death, as some older subjects are self-selecting for subjects who have not died. This will mean that older subjects are, on average, healthier than younger subjects.

It is important to note that in this field of the UK Biobank, a distinction between type I and type II diabetes is not made. However, as type 2 diabetics constitutes approximately 90% of diabetics in the UK population [35], it is likely that the differences observed here are driven by type 2 diabetes.

This study is not without limitations, and work is ongoing to improve the semi-automated analysis process and the MRI technique used to measure PDFF. Furthermore the PDFF measurements used in this study were derived from MRI images captured using the DIXON technique [20,21], however the IDEAL method [22] is now considered a more robust and accurate method for measuring liver PDFF, therefore the acquisition sequences used by the UKBiobank have been altered to reflect this.

There are many more cofactors that are known or postulated to affect liver steatosis, for example ethnicity, but this cohort does not have sufficient numbers to allow detailed analysis—95.8% of the cohort is ethnically white. More individuals are required to identify trends in phenotypes that have less clear effects, such as ethnicity. As the imaging study progresses, there will be an opportunity to investigate whether previously reported associations with these and other factors are reproduced in the UK Biobank imaging cohort.

## Conclusions

This study has characterised the epidemiology of hepatic steatosis in the UK Biobank cohort. We have identified the normal range for the whole cohort, and a healthy (BMI < 25 kg/m^2^, no diabetes diagnosed by doctor) subset. This demonstrates that analysis of hepatic steatosis (PDFF) in a large cohort by MRI is feasible and can be completed with low failure rates (~3.5%). The median PDFF in the whole cohort Is 2.11%, and the 95% upper limit of PDFF is 5.21% in a healthy subset of the cohort, which is consistent with previous studies.

We observed significant correlations of PDFF with age, sex, BMI, weight gain, high blood pressure and diabetes. This is consistent with the current understanding of NAFLD as a metabolic disorder. This analysis also reveals simple thresholds to stratify subjects for higher risk PDFF of greater than 5%. Further analysis is required to identify whether genetic factors or other phenotypic measures within the UK Biobank can contribute to prediction of PDFF with even higher accuracy.

With future follow up of incident diseases in the UK Biobank population, the cross-sectional measures of PDFF will enable more accurate estimates of prospective risks of fatty liver disease (NAFLD) for the associated phenotypes of NASH, hepatic fibrosis and hepatocellular carcinoma. These conditions are on the rise, and are expected to have a significant impact on public health in the coming decades.

## Supporting information

S1 File Full UK Biobank acknowledgements. (DOCX)

S1 Table PDFF quartiles for BMI ranges. 20 participants had a missing BMI value.
(DOCX)

S2 Table Coefficients in linear model for predicting PDFF, using a log transformation on PDFF. (DOCX)

S3 Table Correlation coefficients between the regression coefficients used in the linear model. (DOCX)

S4 Table Confusion matrix showing the interaction between diabetes and proton density fat fraction. Half of individuals with Diabetes had at least 5.5% PDFF.
(DOCX)

S5 Table Diabetes and BMI as a predictor of PDFF. (DOCX)

S6 Table BMI as a predictor of liver fat. (DOCX)

S7 Table Ability of BMI and PDFF to predict diabetes. (DOCX)
