# Correction: Characterisation of liver fat in the UK Biobank cohort

**DOI:** 10.1371/journal.pone.0176867

**Published:** 2017-04-26

**Authors:** Henry R. Wilman, Matt Kelly, Steve Garratt, Paul M. Matthews, Matteo Milanesi, Amy Herlihy, Michael Gyngell, Stefan Neubauer, Jimmy D. Bell, Rajarshi Banerjee, E. Louise Thomas

The seventh author's name is spelled incorrectly. The correct name is: Michael Gyngell.
