# Supplementary material for: Characterisation of liver fat in the UK Biobank cohort
Source: PLoS One. 2017 Feb 27;12(2):e0172921. doi: 10.1371/journal.pone.0172921 (PMC5328634; doi:10.1371/journal.pone.0172921)
Supplement: S1 Table — 20 participants had a missing BMI value. (DOCX) [file pone.0172921.s002.docx]

**S1 Table. PDFF quartiles for BMI ranges.** 20 participants had a missing BMI value

| All | BMI < 20 | BMI >= 20  BMI < 25 | BMI >= 25  BMI < 30 | BMI >= 30  BMI < 35 | BMI < 35 |
| --- | --- | --- | --- | --- | --- |
| 1^st^ quartile | 0.79 | 1.02 | 1.59 | 2.22 | 4.07 |
| Median | 0.98 | 1.36 | 2.54 | 4.39 | 7.33 |
| 3^rd^ quartile | 1.22 | 2.13 | 4.81 | 8.72 | 13.48 |
| Count | 133 | 1646 | 1952 | 657 | 209 |
