# Supplementary material for: Characterisation of liver fat in the UK Biobank cohort
Source: PLoS One. 2017 Feb 27;12(2):e0172921. doi: 10.1371/journal.pone.0172921 (PMC5328634; doi:10.1371/journal.pone.0172921)
Supplement: S2 Table — (DOCX) [file pone.0172921.s003.docx]

**S2 Table. Coefficients in linear model for predicting PDFF, using a log transformation on PDFF.** Beta is the effect size, SE the standard error on the effect size estimate. P is the probability (i.e. p-value) that the phenotype is not correlated with PDFF.

|  | Beta | SE | P |
| --- | --- | --- | --- |
| Age | 0.0069 | 0.0015 | 6.36e-6 |
| BMI | 0.0942 | 0.0026 | <2e-16 |
| Male | 0.1894 | 0.0217 | <2e-16 |
| High blood pressure | 0.1199 | 0.0253 | 2.18e-6 |
| Weight gain | 0.1242 | 0.0268 | 3.51e-6 |
| Angina | -0.1092 | 0.0740 | 0.14 |
| Diabetes | 0.3516 | 0.0511 | 7.13e-12 |
