# Supplementary material for: Characterisation of liver fat in the UK Biobank cohort
Source: PLoS One. 2017 Feb 27;12(2):e0172921. doi: 10.1371/journal.pone.0172921 (PMC5328634; doi:10.1371/journal.pone.0172921)
Supplement: S3 Table — (DOCX) [file pone.0172921.s004.docx]

**S3 Table. Correlation coefficients between the regression coefficients used in the linear model.**

|  | BMI | Age | Male | Angina | High Blood Pressure | Weight gain | Diabetes |
| --- | --- | --- | --- | --- | --- | --- | --- |
| BMI | 1 | 0.04 | -0.12 | -0.05 | -0.18 | -0.28 | -0.14 |
| Age |  | 1 | -0.04 | -0.07 | -0.19 | 0.10 | -0.04 |
| Male |  |  | 1 | -0.04 | -0.06 | 0.16 | -0.03 |
| Angina |  |  |  | 1 | -0.05 | 0.00 | -0.05 |
| High Blood Pressure |  |  |  |  | 1 | 0.01 | -0.10 |
| Weight gain |  |  |  |  |  | 1 | 0.02 |
| Diabetes |  |  |  |  |  |  | 1 |
