# Supplementary material for: Characterisation of liver fat in the UK Biobank cohort
Source: PLoS One. 2017 Feb 27;12(2):e0172921. doi: 10.1371/journal.pone.0172921 (PMC5328634; doi:10.1371/journal.pone.0172921)
Supplement: S4 Table — Half of individuals with Diabetes had at least 5.5% PDFF. (DOCX) [file pone.0172921.s005.docx]

**S4 Table. Confusion matrix showing the interaction between diabetes and proton density fat fraction.** Half of individuals with Diabetes had at least 5.5% PDFF.

|  | PDFF <= 5.5 % | PDFF > 5.5% |  |
| --- | --- | --- | --- |
| No diabetes | 3542 | 803 | NPV = 0.82 |
| Diabetes | 113 | 113 | PPV = 0.50 |
|  | Specificity = 0.97 | Sensitivity = 0.12 |  |
