# Supplementary material for: Characterisation of liver fat in the UK Biobank cohort
Source: PLoS One. 2017 Feb 27;12(2):e0172921. doi: 10.1371/journal.pone.0172921 (PMC5328634; doi:10.1371/journal.pone.0172921)
Supplement: S5 Table — (DOCX) [file pone.0172921.s006.docx]

**S5 Table. Diabetes and BMI as a predictor of PDFF.**

|  | PDFF <= 5.5 % | PDFF > 5.5% |  |
| --- | --- | --- | --- |
| No diabetes or BMI < 25 kg/m^2^ | 3587 | 809 | NPV = 0.82 |
| Diabetes & BMI >= 25 kg/m^2^ | 84 | 106 | PPV = 0.56 |
|  | Specificity = 0.98 | Sensitivity = 0.12 |  |

Confusion matrix showing the number of individuals that have more than 5.5% liver fat, and both BMI greater than 25 kg/m^2^ and Diabetes. More than half the individuals with both an elevated BMI and with Diabetes have an elevated PDFF, giving a PPV of 0.56
