# Supplementary material for: Characterisation of liver fat in the UK Biobank cohort
Source: PLoS One. 2017 Feb 27;12(2):e0172921. doi: 10.1371/journal.pone.0172921 (PMC5328634; doi:10.1371/journal.pone.0172921)
Supplement: S6 Table — (DOCX) [file pone.0172921.s007.docx]

**S6 Table*.* BMI as a predictor of liver fat.** Confusion matrix showing the number of individuals that have more than 5.5% liver fat, and a BMI greater than 25 kg/m^2^. Very few individuals with BMI under 25 kg/m^2^ have PDFF above 5.5%, resulting in a high negative predictive value (NPV) of 0.95. However, many individuals with BMI above 25 kg/m^2^ do not have elevated PDFF, hence a BMI threshold of 25 kg/m^2^ has a low positive predictive value (NPV) of 0.30.

|  | PDFF <= 5.5 % | PDFF > 5.5% |  |
| --- | --- | --- | --- |
| BMI < 25 kg/m^2^ | 1696 | 83 | NPV = 0.95 |
| BMI >= 25 kg/m^2^ | 1983 | 835 | PPV = 0.30 |
|  | Specificity = 0.46 | Sensitivity = 0.91 |  |
