# Supplementary material for: Characterisation of liver fat in the UK Biobank cohort
Source: PLoS One. 2017 Feb 27;12(2):e0172921. doi: 10.1371/journal.pone.0172921 (PMC5328634; doi:10.1371/journal.pone.0172921)
Supplement: S7 Table — (DOCX) [file pone.0172921.s008.docx]

**S7 Table. Ability of BMI and PDFF to predict Diabetes.** The combination of BMI and PDFF has a high sensitivity for predicting Diabetes. Only 26 of the 226 diabetics have both a BMI less than 25 kg/m2 and a PDFF less than 5.5%

|  | No Diabetes | Diabetes |  |
| --- | --- | --- | --- |
| PDFF <= 5.5% and BMI < 25 kg/m^2^ | 1651 | 29 | NPV = 0.98 |
| PDFF >= 5.5% or BMI >= 25 kg/m^2^ | 2687 | 197 | PPV = 0.07 |
|  | Specificity = 0.38 | Sensitivity = 0.87 |  |
